# Possible contribution of chronobiology to cardiovascular health

**DOI:** 10.3389/fphys.2013.00409

**Published:** 2014-01-20

**Authors:** Miho Sato, Takahiro Matsuo, Henry Atmore, Makoto Akashi

**Affiliations:** ^1^The Research Institute for Time Studies, Yamaguchi UniversityYamaguchi, Japan; ^2^Department of Anglo-American Studies, Kobe City University of Foreign StudiesKobe, Japan

**Keywords:** circadian, clock gene, non-invasive method, cardiovascular diseases, hair follicle

## Abstract

The daily variations found in many aspects of physiology are collectively known as circadian rhythm (from “circa” meaning “about” and “dien” meaning “day”). Circadian oscillation in clock gene expression can generate quantitative or functional variations of the molecules directly involved in many physiological functions. This paper reviews the molecular mechanisms of the circadian clock, the transmission of circadian effects to cardiovascular functions, and the effects of circadian dysfunction on cardiovascular diseases. An evaluation of the operation of the internal clock is needed in clinical settings and will be an effective tool in the diagnosis of circadian rhythm disorders. Toward this end, we introduce a novel non-invasive method for assessing circadian time-regulation in human beings through the utilization of hair follicle cells.

## Introduction

Circadian variations are observed in many physiological functions; they are generated autonomously by an internal circadian clock. The circadian period in human subjects is slightly longer than 24 h. Light and food intake have been found to be effective factors in phase adjustment to the environmental cycle. The circadian clock is composed of transcriptional feedback loops between clock genes that regulate quantitative variations in functionally related genes in almost all cells in the human body. Models constructed from studies of mouse subjects have shown that dysfunctions of the circadian clock are a risk factor in certain cardiovascular conditions. In order to study the relations between cardiovascular problems and the circadian clock in man, methods to elucidate the operation of the clock in human subjects should be improved. In this review, we will survey the risks associated with dysfunction of the circadian clock suggested by the mouse studies. We will then propose possible approaches to, and future improvements in, the assessment of the operation of the circadian clock in humans.

## The circadian clock and modern life

**Figure 1:**
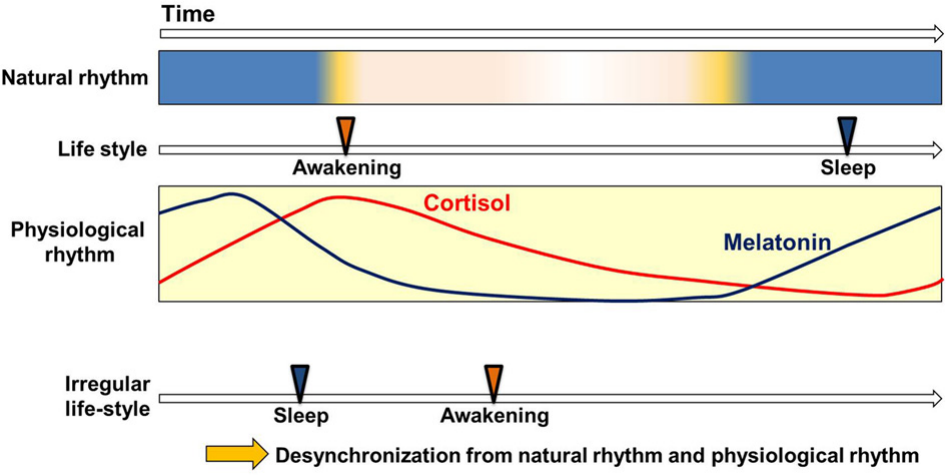
**Circadian clock and life style**. Circadian variation of physiological functions is set to synchronize with the natural environment. This enables the smooth operation of physiological functions in harmony with the basic rhythms (Weitzman et al., 1971; Lewy and Markey, 1978; Arendt, 2005; Haus, 2007; Benloucif et al., 2008) and cycles of nature (light-dark) and with the correlated but not always continuous rhythms and cycles of human life-style (waking-sleep). Irregular life-styles bring about the desynchronization of physiological rhythms from other sources of cyclicity.

Most organisms, both diurnal and nocturnal, adapt their physiological functions to the basic natural 24-h light-dark cycle (Hastings et al., 2008). Human physiology prepares for daytime-activities by up-regulating body temperature and blood pressure, and prepares for sleep by down-regulating them (Aschoff, 1965). Circadian variations are also found in endocrine secretions, metabolic processes, neuronal activities, and immune functions (Weitzman et al., 1971; Lewy and Markey, 1978; Haus, 2007; Bass, 2012; Scheiermann et al., 2013). In all of these cases they operate without external time cues (Figure 1). That is to say, the circadian clock can generate circadian variations autonomously (Mills, 1964; Aschoff, 1965), enabling the efficient readying and discharge of physiological functions, to the organism's great advantage.

Because the circadian period in human subjects is slightly longer than 24 h, the phase must be adjusted every day to prevent the desynchronization of inner rhythm and the environmental cycle. The light and food intake have been found effective for the entraining of this adjustment. However, disordered light conditions and irregular meal times create anomalies in the synchronization of internal biorhythm with the external cycle. Many aspects of modern life uncouple our physiological rhythms from our natural environment: one does not have to travel to experience the equivalent of jet-lag. An important focus for current research is the connection between certain modern “life style” pathologies and the circadian clock.

## A clock in every cell

The circadian clock is composed of transcriptional feedback loop between clock genes (Okamura et al., 2010; Takeda and Maemura, 2011). The autonomous regulation of clock gene expression is thought to create a ~24 h oscillation which is transmitted to produce parallel physiological fluctuations. Interestingly, clock genes oscillate not only in the suprachiasmatic nucleus (SCN), located in the diencephalon, but in almost all peripheral cells (Yamazaki et al., 2000). Circadian rhythmicity has been detected in single-cells cultured *in vitro*. Differences in the individual periods of individual cells need to be phase adjusted to avoid internal desynchronization (Nagoshi et al., 2004), which would lead to discordance in physiological rhythm. This suggests a function for the SCN, to entrain peripheral clocks as the internal pacemaker.

There are about 20 clock genes in mammals, but the core functioning genes amount to 7, of 4 types: *Period* (abbreviated as *Per; Per1, Per2*, and *Per3*), *Cryptochrome* (abbreviated as *Cry*; *Cry1* and *Cry2*), *Clock* and *Bmal1* (Lowrey and Takahashi, 2011, Figure 2). PER and CRY form a hetero dimer and work as suppressors. CLOCK and BMAL1 activate transcription of *Per* through the *cis* element E-boxes. PER decreases gradually as a result of repression, and the CLOCK/BMAL1 heterodimer activates the expression again. This autonomous feedback repeats approximately every 24 h. The system maintains stable periodicity through reinforcement by other transcriptional regulators, such as ROR, REV-ERB, DBP, and E4BP4.

**Figure 2:**
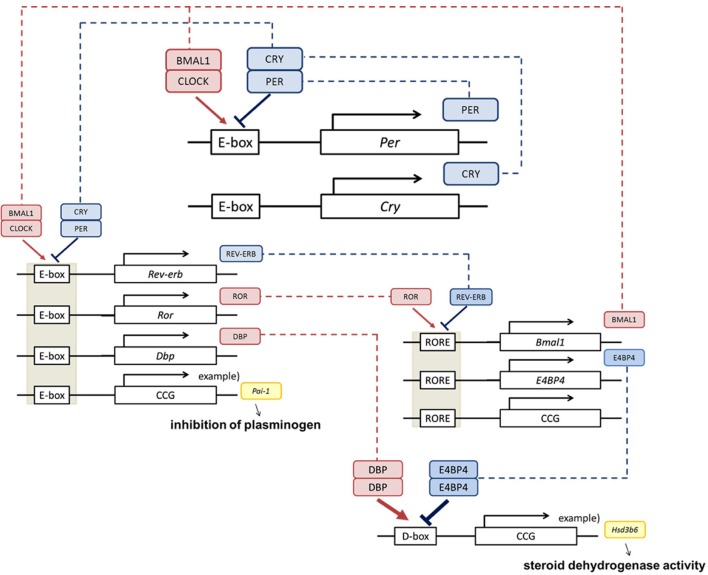
**Clock genes and physiological function**. The core clock is composed by the *Per, Cry, Bmal1*, and *Clock* clock genes (Okamura et al., 2010; Takeda and Maemura, 2011). BMAL1 and CLOCK activate transcriptional levels through E-boxes, and CRY and PER suppress this activity. *Cis* elements such as RORE and D-box can be regulated by ROR, REV-ERB, DBP, and E4BP4, and multilayered rigid circadian rhythms are ticked down. This negative feedback loop produces the rhythm of transcription. These regulations are transmitted via transcriptional fluctuations of clock controlled genes (ccg) such as *plasminogen activator inhibitor-1* (*Pai-1*) (Maemura et al., 2000; Haus, 2007), *type VI 3 beta-hydroxyl-steroid dehydrogenase* (*Hsd3b6*) (Bass, 2012).

## Circadian clock and cardiovascular function

There is an increasing body of evidence that the increasing intensity of cardiovascular events in the morning hours (Muller et al., 1985; Paschos and FitzGerald, 2010), indicating circadian rhythm is also found in cardiovascular functions. Studies of healthy subjects show a clear decrease in endothelial function in the early morning, implying that this plays a role in inducing the mechanisms of the morning peak (Otto et al., 2004). Blood pressure, heart rate, and the operation of the renin-angiotensin-aldosterone system (RAAS) have been shown to increase in the morning (Williams et al., 1972; Mancia et al., 1983). Thrombotic tendencies are elevated early in the morning along with platelet aggregation and the fibrinolytic system (Fearnley et al., 1957; Tofler et al., 1987). Vascular resistance and sympathetic activation are also evident before waking (Panza et al., 1991). These morning bias may be related to ischemic diseases such as subarachnoid hemorrhage, cerebral infarction, vascular disease of the brain, angina, and myocardial infarction (Muller et al., 1985; Paschos and FitzGerald, 2010).

The circadian clock is operative in both endothelial and smooth muscle cells (Reilly et al., 2007). The clock gene regulates the quantitative variation of ccg's (clock controlled genes)—such as *plasminogen activator inhibitor-1* (*Pai-I*), *type VI 3 beta-hydroxyl-steroid dehydrogenase* (*Hsd3b6*)—which are specifically expressed in adipocyte and adrenal zona glomerulosa cells respectively and directly operate inhibition of plasminogen or steroid dehydrogenase activity respectively (Maemura et al., 2000; Schoenhard et al., 2003; Doi et al., 2010, Figure 2). Global analysis of the aorta has demonstrated that about 300 relevant genes, including vascular function related genes, display daily fluctuations (Rudic et al., 2005). To elucidate the role of core-clock genes within the circulatory system, mouse models deficient for specific core-clock genes have been evaluated. Disordered amplitude, period and phase of blood pressure and heart rate were reported, although there are phenotypical variations in these effects depending on which gene was deactivated (Curtis et al., 2007; Vukolic et al., 2010; Bonny et al., 2013; Richards and Gumz, 2013). The balance of renal excretion/reabsorption of water and electrolytes is considered a major factor for blood pressure. Analysis with Per1 KO or Clock mutant mice, in which blood pressure is significantly reduced, suggests that several key molecules are controlled by the adrenal clock (Zuber et al., 2009; Nikolaeva et al., 2012; Stow et al., 2012). In hypertensive humans, abnormal circadian expression of PER1 has been found by transcriptome analysis (Marques et al., 2011). In *Bmal1*-knockout or *Clock* mutant mice, increased pathological vascular remodeling and injury together with disrupted thrombosis were observed (Westgate et al., 2008; Anea et al., 2009). In the aorta of these mice, subsequent endothelial dysfunction led to significantly low activity of AKT (Anea et al., 2009). In endothelial cells from *Per2* mutant mice, AKT signaling and vascular senescence were increased indicating AKT-dependent senescence (Wang et al., 2008). Last, in the aortic ring of *Per2* mutant mice, impaired endothelial-dependent relaxations to the administration of acetylcholine were observed (Viswambharan et al., 2007). These studies conclusively show that clock genes fulfill critical roles in the regulation of cardiovascular functions.

## Circadian clock and arteriosclerosis

Recent studies have found that dysfunction of the circadian clock represents the risk factor for arteriosclerosis. A study published in 2005 showed that *Clock* mutant mice manifest elevated blood glucose levels, lipid abnormalities, and obesity (Turek et al., 2005). Regarding the last of these, BMAL1 regulates circadian fluctuations in genes involved in lipid metabolism, indicating a connection between the periodicity of meal times and obesity (Shimba et al., 2005). Deficiency in *Clock* or *Bmal1* triggers defects in the size of the pancreatic islet, and reduced insulin secretion (Marcheva et al., 2010, Figure 3). On the other hand, knock down of *Cry1* and *Cry2* induces up-regulation of blood glucose levels and insulin resistance, so CRY is considered to be a repressor of gluconeogenesis (Zhang et al., 2010). Moreover, in *Cry1*, *2* double-deficient mice, abnormally high aldosterone synthesis is indicated, tending toward salt-sensitive hypertension (Doi et al., 2010, Figure 4). Still, a direct relation between the circadian clock and arteriosclerosis has not as yet been demonstrated. However, taking a holistic view of the relations between arterial functions, the circadian clock, and the up-regulation of risk factors of arterial sclerosis caused by clock gene deficiency, it appears to be true that desynchronization between the circadian clock and the organism's life-style worsens the situation.

**Figure 3:**
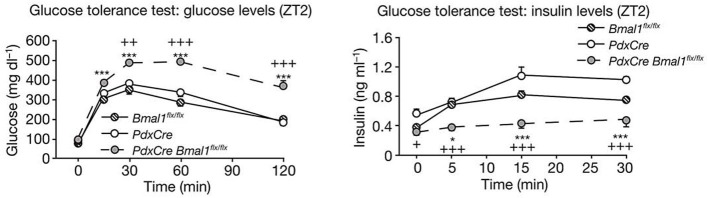
**Clock genes and diabetes**. Glucose levels (left panel) and insulin levels (right panel) during glucose tolerance tests in pancreas-specific Bmal1-deficient mice (*PdxCre Bmal1^flx/flx^*). *PdxCre* and *Bmal1^flx/flx^* mice are negative controls for the experiments. *Bmal1* knockout mice displayed decreased insulin secretion and higher glucose levels after glucose administration. The figures are adapted from Macmillan Publishers Ltd: Nature, Marcheva et al. (2010), copyright 2010. Data were analysed by one-way ANOVA and asterisks denote significance between *Bmal1^flx/flx^* and *PdxCre Bmal1^flx/flx^*, and plus symbols denote significance between *PdxCre* and *PdxCre Bmal1^flx/flx^*. ^*^p = 0.05; ^***^p = 0.001.

**Figure 4:**
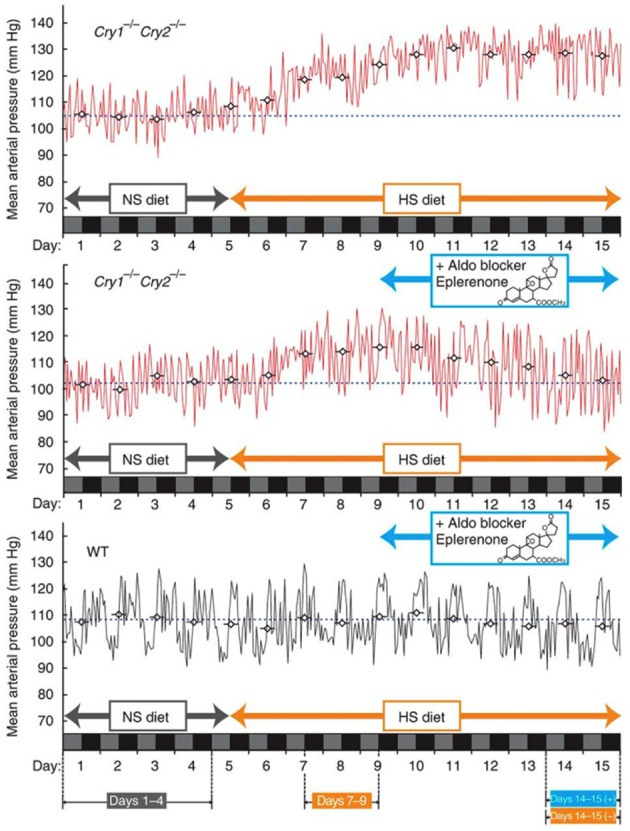
**Clock genes and hypertension**. Fifteen-day mean arterial blood pressure recorded under high-salt diet conditions. Mice lacking the core clock components *Cryptochrome1* (*Cry1*) and *Cryptochrome2* (*Cry2*) (*C*ry1^−/−^ Cry2^−/−^, upper panel) show salt-sensitive hypertension, while the wild type (WT) doesn't. This hypertension can be avoided with the aldosterone inhibitor, Eplerenone. The figures are adapted from Macmillan Publishers Ltd: Nature Medicine, Doi et al. (2010), copyright 2010.

## Chronobiological approaches to cardiovascular health

As mentioned above, dysfunction of the circadian clock may be a potential risk factor for cardiovascular diseases. If additional studies in actual human disorders reveal that this dysfunction is often observed in patients, clinical methods will be required to diagnose whether this is due to autonomous clock dysfunction (endogenous factors) or to lifestyle habits (environmental factors). We therefore propose a two-step diagnosis: the first step is biopsy-based and the second is tissue culture-based. The data obtained from the first step covers both endogenous and environmental factors, whereas the second basically covers only endogenous factors such as individual differences in genetic or epigenetic background. Careful comparison between the results obtained from these two steps would provide the information necessary to carry out appropriate treatment for individual circadian disorders.

The first step will enable diagnosis of what might be called social jet lag (desynchronization between internal and external cycles). Although melatonin measurement has been traditionally adopted in such diagnoses, and serum metabolites more recently utilized as circadian markers, these are likely affected by light- or feeding-induced masking, and accordingly require a controlled environment within the laboratory. More fundamentally, the investigation of the circadian pacemaker should require the examination of circadian clock gene expression, since the core clock is composed of cell-autonomous transcriptional feedback loops. Several reports have indicated that it is possible to measure clock gene expression through biopsies in human subjects. For example, human visceral and subcutaneous adipose tissues (VAT and SAT) (Gómez-Abellán et al., 2008, 2012a; Otway et al., 2011; Hernandez-Morante et al., 2012) are used to compare differences in clock gene expression between, variously, obese and diabetic subjects (Otway et al., 2011), males and females (Gómez-Abellán et al., 2012a), and premenopausal and menopausal women (Hernandez-Morante et al., 2012). Tissue biopsies are powerful tools for obtaining information in research settings, although they would be troublesome for patients in clinical settings. The circadian phase displayed in our recently established method utilizing hair follicle cells is similar to that displayed in leukocytes (Watanabe et al., 2012). For this reason we offer it as a potential solution to this difficulty, and a way of moving forward in out-of-lab investigation and diagnosis of human clock mechanisms, although we recognize that it is a method that still requires some technical fine-tuning. Details of the hair-follicle method will be given in the next section of this review-paper.

A report from Brown's group suggests that it is possible to perform the second step using primary culture cells (Brown et al., 2005) and monitoring clock gene expression for autonomous clock dysfunction. They have already succeeded in detecting a correlation between fibroblast period length and the human chronotype (Brown et al., 2008), and also found that there was no difference in peripheral clock properties between young and old subjects (Pagani et al., 2011). Interestingly, this group has shown that blind individuals showed on average the same fibroblast clock properties as sighted ones in spite of different physiological periods (Pagani et al., 2010). Several applications of this method have been reported from other research groups; primary adipocytes were obtained from VAT and SAT by performing biopsies, and these two types of adipocytes were cultured and compared in terms of expression levels, circadian phases, amplitudes and circadian periods (Gómez-Santos et al., 2009; Garaulet et al., 2011; Gómez-Abellán et al., 2012b).

## Current methods for the detection of the circadian clock in human beings

Though the circadian clock has a deep relationship to the rhythms of everyday human existence, the findings from studies in mice have not been fully replicated in studies of *Homo sapiens*. Since the cloning of the first clock gene in mammals in 1997 (Tei et al., 1997), only a handful of reports examining clock gene activity in man have been published. This is mainly because methods for the detection of circadian clock functions in man is difficult owing to the invasive character of the established experimental procedures. The development of non-invasive, easily-replicated methods is urgent. As mentioned above, almost all cells in the body contain a circadian clock. Therefore, the peripheral clock can be used to evaluate circadian functions because it behaves basically the same as the central clock in the SCN. In 2001, a method using detached oral mucosa was reported (Bjarnason et al., 2001). But when we tried to reproduce these results, most of the specimen-cells were dead and the purified RNA was highly fragmented. This method faces problems that await solution. Later, in 2003, a method using blood taken from a peripheral vein was reported (Boivin et al., 2003). In this case, blood collection every few hours is required, which is a significant hurdle to overcome with human subjects. Additionally, in the case of white blood cells, physical stimuli and time delays due to the processing of cell separation may affect levels of expression of clock genes. So, although modifications to these procedures may avoid these problems, we have not been able to obtain reproducible data from either of them. As a result, we decided to focus our efforts on another source of peripheral tissue.

## Utilization of hair follicles

Living cells must be collected with minimal damage to human subjects. We assumed that a considerable number of cells would be attached to the roots of freshly plucked hair. We then attempted to determine clock-gene expression in cells attached to scalp hair by a real-time PCR method (Akashi et al., 2010, Figure 5).

**Figure 5:**
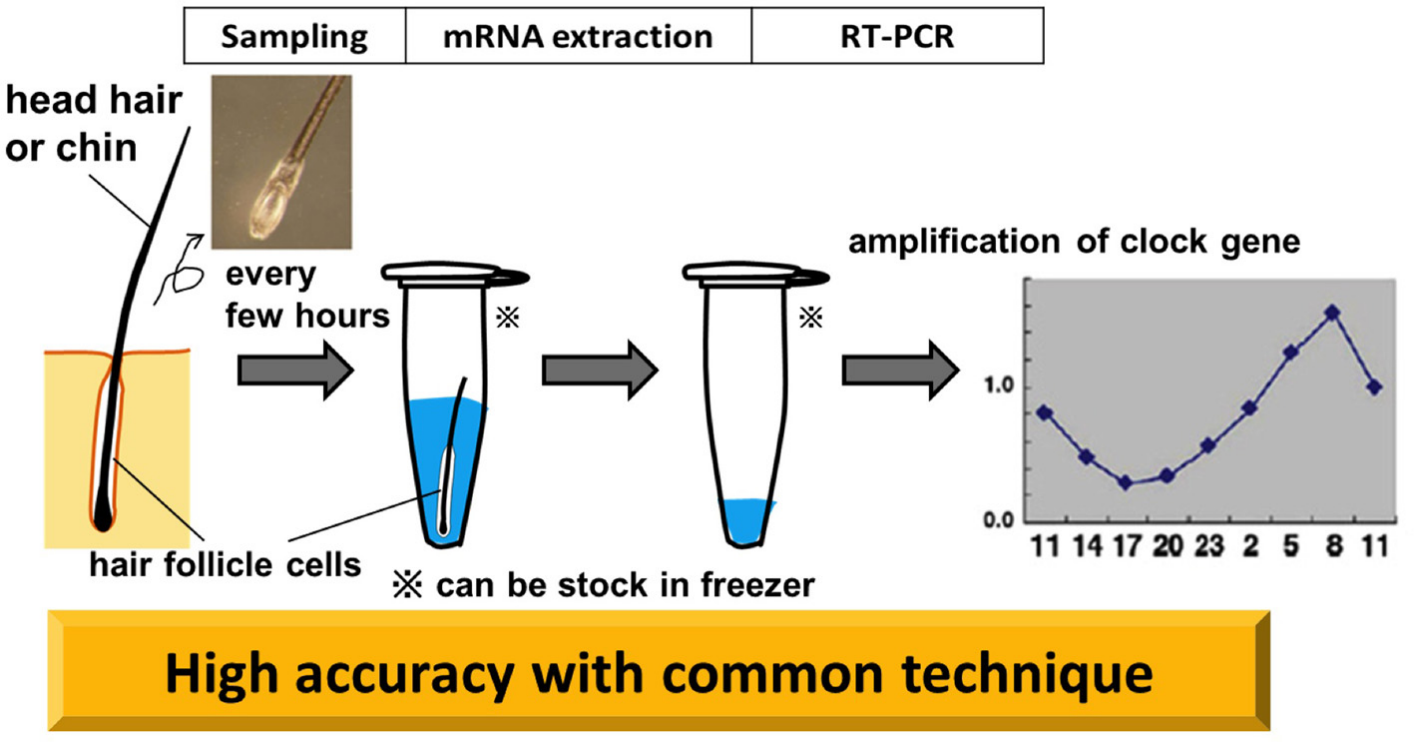
**Non-invasive circadian clock detection with hair follicle cells**. Overview from sampling to quantification by realtime-PCR (RT-PCR). Plucking head hair sharply at the roots with non-slip tweezers in the direction of hair growth tends to extract a good deal of hair follicles. Pre-analysis, samples can be stocked in a deep freezer. The standard protocol for RT-PCR is adopted for the analysis. The graph in this diagram is adapted from Akashi et al. (2010).

Total RNA purified from scalp hair follicle cells exhibits clearly distinguishable absorbance peaks represented by 18S- and 28S-rRNA signals. Advantages of this method are that the quantity of RNA is comparatively high (100 ng of RNA was extracted from each hair follicle), and that the commonly used reverse transcriptional method is applicable (Akashi et al., 2010, Figure 5). Our determination, from this material, of the circadian variation of clock gene expression showed differences from the results obtained from mouse peripheral tissue. For *Bmal1* and *Npas2*, only slight oscillations were detected in human hair follicle cells. This is not an epiphenomenon of the technique, as these results are consistent with previous findings—from DNA microarray analysis—that only *Per3*, *Nr1d1* (*Rev-erbα*), and *Nr1d2* (*Rev-erbβ*) genes could be identified as rhythmically expressed clock genes. Some subjects showed *Per2* and *Dbp* gene oscillations at lower amplitudes, although these did not always display high cosine curve fitting. *Per3, Nr1d1*, and *Nr1d2* genes exhibited circadian fluctuations with good reproducibility in humans, despite the fact that in mice all of these genes are implicated in the regulation of physiological functions, rather than serving as components of the classical core negative feedback loop. The most interesting result is that with most specimens, *Per3* and *Nr1d2* were highly reproducible and manifested a high-fit cosine curve oscillation. While it is not clear why these three genes should fluctuate more widely than the core clock genes, they do meet the criteria for rhythm markers of the circadian clock.

## Reflection of life style in clock gene expression in hair follicles

We examined circadian phase in head hair follicle cells from four subjects who maintain regular life-styles, but with differences in their sleeping patterns (from early to late-risers) (Akashi et al., 2010, Figure 6). Sampling was begun after 10 days of a fixed waking/meal/sleep schedule based on the established life-style of each individual. We expected to see a phase correlation between the life style and the clock gene expression. As expected, circadian fluctuations of *Per3, Nr1d1*, and *Nr1d2* were detected in all subjects and the peak time of expression of those genes also correlated with average waking-times. Next, we compared the clock gene expression phase of liver and kidney cells in mice with that of hair follicle cells in human beings. Interestingly, in both species, *Per3* expression peaked just around waking time (note that the onset of activity occurs in mice immediately after lights-off). This suggests that the phase correlation between behavioral rhythms and circadian clock gene expression is conserved and is independent of whether the animals are diurnal or nocturnal.

**Figure 6:**
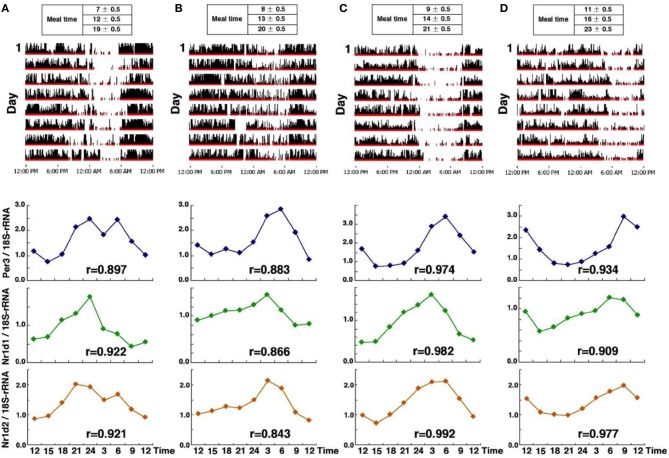
**Clock gene expression from four subjects who maintained a regular lifestyle with a specific phase**. Activity data over an 8-day period (upper panel) and clock gene expression (*Per3, Nr1d1*, and *Nr1d2*) (lower panel) in four healthy individuals **(A–D)**, from the earliest riser on the left **(A)**, to the latest riser on the right **(D)**. Interestingly, in all subjects, *Per3* expression peaked just around waking time and the earliest phase of circadian gene expression was observed in subject **(A)**. The r, correlation coefficient is calculated by cosine curve fitting. The figures are adapted from Akashi et al. (2010).

Next, we examined the phase shift of clock gene expression rhythms in healthy subjects caused by a gradual phase advance of the waking/meal-time/sleeping schedule, by 4 h over a 3 week period (−1 h/5 d) (Akashi et al., 2010, Figure 6). To bolster the effects of the phase advance, the subjects were exposed to ~10,000-lux light for 30 min just after waking. Under these conditions, the average phase advances of clock gene expression rhythms in hair follicle cells ranged from 2.1 to 2.8 h, indicating that a 3-week adaptation period is not sufficient to advance the phase of the molecular clock by 4 h. Phase adjustment to get rid of jet lag is generally believed to proceed at a rate of about 1 h a day, but here we find that the body's accommodation of a 4-h advance is still incomplete after 3 weeks. Though we can't deny possible imperfections in the experimental conditions, for example social stimulus providing time-cues calibrated to the earlier life-style schedule, some factor related to the suddenness of jet-lag etiology might also account for the difference. This unexpectedly slow adjustment is also found in rotating shift workers, to whom we shall now turn.

## Circadian clock gene expression in rotating shift workers

Rotating shift work is symbolic of round-the-clock social/economic conditions in modern Japan, and its incidence is increasing all over the world. Representative types include factory-workers, nurses, security guards, and police officers. For our study of circadian regulation in rotating shift workers we chose subjects employed in an automotive parts factory and investigated the circadian properties of clock-gene expression in their head hair follicle cells, as described in the previous two sections. We analyzed hair follicle cells from 6 subjects in their 20's and 30's (Akashi et al., 2010, Figure 7). All the subjects work 5 successive days a week, alternating between early shifts (0600—1500 h) and late shifts (1500—0000 h) on a weekly basis. This amounts to an, on average, 7 h life-style shift every week, a fairly moderate variation by rotating shift work standards. Scalp hair samples were collected every 3 h on weekends to ascertain the rhythms of clock gene expression, over the course of a 3-week shift rotation, with the 1st week an early shift, the 2nd week a late shift, and the 3rd week an early shift again. An adaptation term of 5 days (from Monday to Friday) was set. The result belied expectations of a drastic phase shift in clock gene expression rhythms for the first rotation. The phase of circadian gene expression was delayed or advanced only by about 2 h, whereas the life style was phase-delayed or advanced by about 7 h (Akashi et al., 2010, Figure 8). A 1-week adaptation was thus not sufficient for completing entrainment in this group of subjects. As mentioned above, it is known that waking-time and meal-times are the most effective entrainers in mice, but it would seem that time adjustment in man takes a great deal more time under similar base conditions. These subjects are living, as it were, in a state of permanent jet lag. It is, from this perspective, hardly surprising that rotating shift workers experience many health problems.

**Figure 7:**
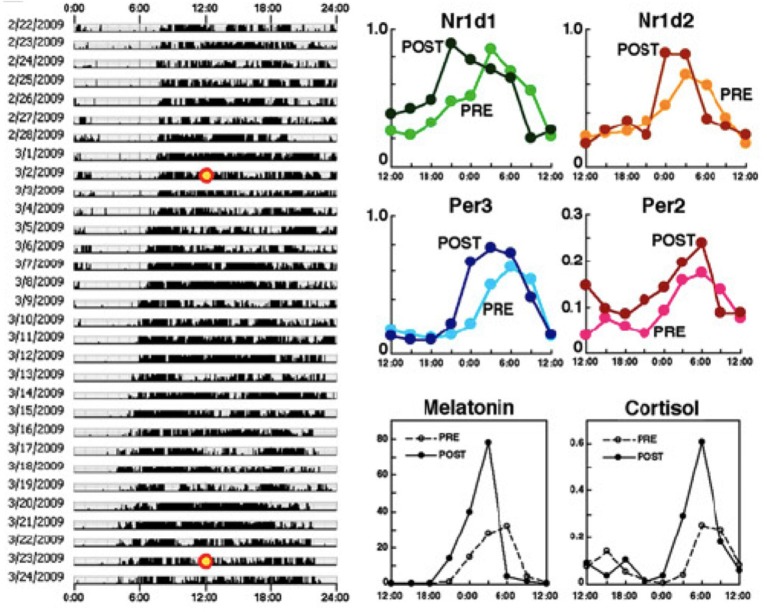
**A 3-week adaptation period is not enough to advance the phase of the molecular clock by 4 h**. Behavioral rhythms (left), rhythms in four clock genes from hair follicles (upper right) and melatonin and cortisol concentrations from saliva (lower right). Specimens were collected every 3 h from the time points marked with a yellow circle (3/2/2009: PRE, 3/23/2009: POST) and compared between PRE and POST. These results indicate that a 3-week adaptation period is not enough to advance the phase of the molecular clock by 4 h. PRE: data before the phase shift; POST: data after the 4-h phase advance of life style rhythms. The figures are adapted from Akashi et al. (2010).

**Figure 8:**
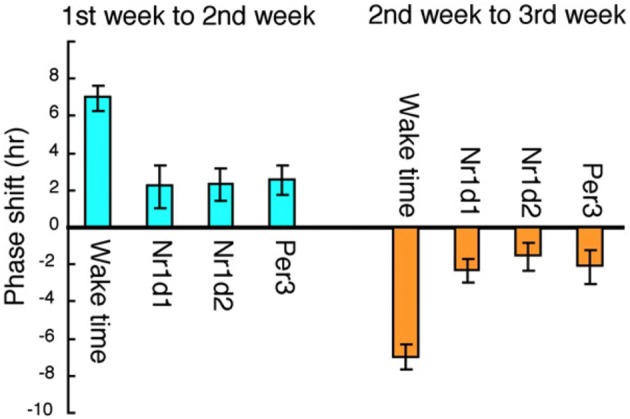
**Correlation between shift-work schedules and clock gene expression in six rotating shift workers**. The average phase shift of waking-time and circadian gene expression was calculated among the six subjects. The phase delay from the 1st to the 2nd week and the phase advance from the 2nd to the 3rd week are shown. The life style was phase-delayed by about 7 h from the 1st to the 2nd week, whereas the phase of circadian gene expression was delayed only by about 2 h. The figures are adapted from Akashi et al. (2010).

It is probably impossible to abolish rotating shift work under current social/economic conditions. Given the increasing prevalence of rotating shift work, the careful coordination of work-schedules taking into consideration lags in the entrainment of workers' circadian clock is necessary from health and incentive perspectives, and may well increase productivity. Concerted institutional support of phase adjustment via manipulation of light-exposure and mealtime schedules may reduce the risk of a number of diseases. Our method can contribute to the optimization of working schedules and provide objective data for productivity gains in services and industries that make heavy use of rotating shifts.

## Future improvements

This paper has outlined what we believe to be a convenient and useful method for assessing circadian clock activity, but we are conscious that there is room for improvement, especially in the area of sample-frequency. Mathematical modeling involving the calculation of the cosine curve with data from 3 different time points of the expression levels of *Per3, Nr1d1*, and *Nr1d2*, suggests one way forward. From this data, compared between specimens, we were able to predict the time phase within a ± 1.5 h time-range.

Previous findings for healthy individuals suggest that the amplitude and level of clock gene expression rhythm remains within a certain range for all individuals. A cosine curve of the average amplitude and level of expression for healthy individuals was therefore defined as the standard curve, and the rhythm of expression in all healthy individuals was assumed to follow it. In three-point phase prediction, phases were determined by the least-squares method to fit the standard curve as closely as possible and the errors were within 1.5 h in most cases. It is likely that we could reduce the parameters by using a standard curve fitted to phase prediction

Note that high quality-RNA can be obtained from both head or chin hair follicles, and more easily reproducible results can be obtained from the collection of smaller numbers of chin hairs than of head hairs. In the current state of the method, in the case of men, 10 head hairs or 5 chin hairs required for one time point. For women, this same number of hairs is usually sufficient, although more are required in some cases. Considering our recent analysis from single head hair (data not shown) formed clear rhythm, 1 head hair may be sufficient for the phase assessment, though levels of clock genes expression in hair follicles are reported to differ from different hair follicle stage in mice (Lin et al., 2009). However, all specimens must be carefully evaluated, as some people have fewer cells attached to their hair-strands than do others. Also, yield may be increased by improving sampling methods, the protocol for RNA extraction, selection of the reverse-transcription enzyme, and finally, re-consideration of the optimal primer and probe for RT-PCR.

## Conclusions

Circadian clock assessment utilizing hair follicles is applicable to a number of current research projects (Watanabe et al., 2012), especially—as we have shown in this paper—studies of the effect of the working environment on fundamental biorhythms. Applications seem feasible in the fields of chronopharmacology and chronotherapy. Notwithstanding the wide range of modern life-styles, this method promises to provide objective information on phase shifts and lags in subjects requiring medication such as under out-of-lab conditions for a fighter (Okamoto et al., 2013) and equine animals (Watts et al., 2012).

Melatonin has been the main marker for the evaluation of internal circadian time up to now (Arendt, 2005; Benloucif et al., 2008). Though this is a distinct marker for the functioning of the central clock, the hair follicle cell method is a powerful tool for the detection of activity in the peripheral clock itself (that is, organism-wide clock gene expression rhythm). Melatonin synthesis is decreased by light (Lewy et al., 1980), and melatonin secretion levels are different between individuals (Arendt, 1998); assessing the peripheral clock doesn't face problems like this. This is a considerable advantage in experimental studies under out-of-laboratory conditions. Moreover, because there are multiple clock genes it will be possible to reduce sampling time by careful mathematical modeling. In combination with the data provided by melatonin detection studies a much wider understanding of the phenomena in question will be obtained.

The ultimate goal is to put this method to practical use in improving human health, specifically the effective treatment and/or prevention of sleep disorders and psychiatric illness stemming from biorhythmic disorder.

### Conflict of interest statement

The authors declare that the research was conducted in the absence of any commercial or financial relationships that could be construed as a potential conflict of interest.
